# Diagnostic and prognostic value of cerebrospinal fluid SNAP-25 and neurogranin in Creutzfeldt-Jakob disease in a clinical setting cohort of rapidly progressive dementias

**DOI:** 10.1186/s13195-023-01300-y

**Published:** 2023-09-08

**Authors:** Giuseppe Mario Bentivenga, Simone Baiardi, Andrea Mastrangelo, Corrado Zenesini, Angela Mammana, Barbara Polischi, Sabina Capellari, Piero Parchi

**Affiliations:** 1 Department of Biomedical and Neuromotor Sciences (DiBiNeM), University of Bologna, Bologna, Italy; 2 IRCCS, Istituto delle Scienze Neurologiche di Bologna, Programma Neuropatologia delle Malattie Neurodegenerative, Bologna, Italy

**Keywords:** Cognitive Disorders, Dementia, Prion disease, SNAP-25, Neurogranin, Tau, Neurofilament, Synapses, Creutzfeldt-Jakob

## Abstract

**Background:**

The levels of synaptic markers synaptosomal-associated protein 25 (SNAP-25) and neurogranin (Ng) have been shown to increase early in the cerebrospinal fluid (CSF) of patients with Creutzfeldt-Jakob disease (CJD) and to have prognostic potential. However, no validation studies assessed these biomarkers' diagnostic and prognostic value in a large clinical setting cohort of rapidly progressive dementia.

**Methods:**

In this retrospective study, using commercially available immunoassays, we measured the levels of SNAP-25, Ng, 14–3-3, total-tau (t-tau), neurofilament light chain (NfL), and phospho-tau181 (p-tau) in CSF samples from consecutive patients with CJD (*n* = 220) or non-prion rapidly progressive dementia (np-RPD) (*n* = 213). We evaluated and compared the diagnostic accuracy of each CSF biomarker and biomarker combination by receiver operating characteristics curve (ROC) analyses, studied SNAP-25 and Ng CSF concentrations distribution across CJD subtypes, and estimated their association with survival using multivariable Cox regression analyses.

**Results:**

CSF SNAP-25 and Ng levels were higher in CJD than in np-RPD (SNAP-25: 582, 95% CI 240–1250 vs. 115, 95% CI 78–157 pg/ml, *p* < 0.0001; Ng: 841, 95% CI 411–1473 vs. 390, 95% CI 260–766 pg/ml, *p* < 0.001). SNAP-25 diagnostic accuracy (AUC 0.902, 95% CI 0.873–0.931) exceeded that of 14–3-3 (AUC 0.853, 95% CI 0.816–0.889), t-tau (AUC 0.878, 95% CI 0.845–0.901), and the t-tau/p-tau ratio (AUC 0.884, 95% CI 0.851–0.916). In contrast, Ng performed worse (AUC 0.697, 95% CI 0.626–0.767) than all other surrogate biomarkers, except for NfL (AUC 0.649, 95% CI 0.593–0.705). SNAP-25 maintained a relatively high diagnostic value even for atypical CJD subtypes (AUC 0.792, 95% CI 0.729–0.854). In Cox regression analyses, SNAP-25 levels were significantly associated with survival in CJD (hazard ratio [HR] 1.71 95% CI 1.40–2.09). Conversely, Ng was associated with survival only in the most rapidly progressive CJD subtypes (sCJD MM(V)1 and gCJD M1) (HR 1.81 95% CI 1.21–2.93).

**Conclusions:**

In the clinical setting, CSF SNAP-25 is a viable alternative to t-tau, 14–3-3, and the t-tau/p-tau ratio in discriminating the CJD subtypes from other RPDs. Additionally, SNAP-25 and, to a lesser extent, Ng predict survival in CJD, showing prognostic power in the range of CSF t-tau/14–3-3 and NfL, respectively.

**Supplementary Information:**

The online version contains supplementary material available at 10.1186/s13195-023-01300-y.

## Background

Creutzfeldt-Jakob disease (CJD) belongs to the prion disease, a group of rare neurodegenerative disorders related to prion protein (PrP) misfolding. CJD is highly heterogeneous with variable etiology (sporadic, genetic, and acquired forms) and phenotypic expression. Sporadic CJD (sCJD), the most common form, comprising about 85% of cases, includes six major clinicopathological subtypes that are primarily determined by the genotype at the polymorphic codon 129 (encoding methionine, M, or valine, V) of the prion protein gene (*PRNP*) and the type (1 or 2) of misfolded PrP (PrP^Sc^) accumulating in the brain (e.g., MM1, VV1, MM2, etc.). About 35% of sCJD cases show mixed phenotypic features due to the co-occurrence of PrP^Sc^ types 1 and 2 and are classified accordingly [1–3]. A genetic form (gCJD) linked to pathogenic *PRNP* mutations accounts for 10–15% of cases. Notably, despite the associated pathogenic mutations, the type of PrP^Sc^ (1, 2 or intermediate size [“i”]) and the codon 129 genotype of *PRNP* mutated allele mainly determine the clinicopathological phenotype of the prevalent gCJD subtypes (e.g., M1, M2C, M2T, etc.), as in sCJD [4].

The early distinction of CJD from other rapidly progressive dementias (RPD) remains challenging, given the lack of specificity of the most common clinical presentations. Moreover, the heterogeneity in survival times and disease course among CJD subtypes often hinders the optimal medical management of these patients [5].

Currently, the in vivo identification of patients with prion disease in the context of a rapidly progressive neurological syndrome is achievable with the highest accuracy using the second-generation cerebrospinal fluid (CSF) prion Real-Tine Quaking-Induced Conversion (RT-QuIC) amplification assay [6–13]. However, RT-QuIC has no prognostic value and does not discriminate among CJD subtypes; besides, the limited availability of the recombinant substrate, requiring in-house production by a specialized laboratory, and the lack of adequately standardized procedures limit the consistent use of this assay in the diagnostic work-up of patients with rapidly progressive neurological syndromes. Moreover, many centers still rely only on the first-generation RT-QuIC assay, which has a lower sensitivity than the second-generation protocol [14]. Altogether, these factors justify the continued use of surrogate CSF markers of neurodegeneration in the diagnostic assessment of patients with RPD and their inclusion with diffusion weighted-magnetic resonance imaging (DW-MRI) and prion RT-QuIC in the current diagnostic criteria [11]. Moreover, identifying and validating novel, alternative markers with high diagnostic and prognostic power remain a research priority [11, 14, 15].

Based on the evidence that synaptic loss is an early sign of neurodegeneration in prion disease and even precedes neuronal death, gliosis, and spongiform change [16–20], current research is increasingly focusing on "synaptic" neurodegeneration biomarkers. In this regard, recent studies found that CSF levels of synaptosomal-associated protein 25 (SNAP-25) and neurogranin (Ng), both enriched in synapses, might distinguish CJD from other neurodegenerative diseases accurately and have prognostic potential [21–24].

However, the diagnostic performance of CSF SNAP-25 and Ng has not yet been evaluated in a RPD cohort reflecting the real-world clinical setting. Moreover, no study has yet assessed the distribution of these biomarkers across the whole CJD spectrum. In this study, we measured CSF SNAP-25 and CSF Ng levels in a large RPD cohort comprising both CJD and non-prion RPD (np-RPD) patients and compared their diagnostic accuracy to the one provided by other CSF surrogate neurodegeneration biomarkers. We also assessed the distribution of synaptic biomarkers' CSF concentrations across different CJD clinicopathological subtypes, providing new insights into their heterogeneous cellular and molecular pathology. Eventually, we evaluated the association between CSF SNAP-25 and Ng levels and clinical variables such as disease stage and survival in CJD.

## Methods

### Inclusion criteria

We retrospectively analyzed CSF samples submitted from 2003 to 2022 to the Neuropathology laboratory at the Institute of Neurological Sciences of Bologna, a major reference laboratory for prion disease in Italy. Samples came from patients presenting with RPD and were sent to our center in the suspect of CJD at the time of the lumbar puncture (LP) for diagnostic purposes. We included patients with a definite neuropathological or probable clinical diagnosis and enough CSF to perform the biomarker assays. Our cohort comprised 433 patients, among whom 220 were affected by CJD, and 213 suffered from np-RPD. We measured the concentration of SNAP-25, 14–3-3, t-tau, neurofilament light chain (NfL), and phospho-tau181 (p-tau) in all samples while Ng levels were assessed only in a subgroup of 215 randomly selected patients (122 CJD, 93 np-RPD), as in preliminary analyses the biomarker showed a poor diagnostic accuracy.

The CJD group comprised 183 participants with sCJD (of which 106 with a neuropathological diagnosis and 77 with a clinical diagnosis of probable sCJD according to the current diagnostic criteria [11], all positive by prion RT-QuIC, and 37 patients with a diagnosis of gCJD. sCJD cases with a definite (i.e., neuropathological) diagnosis were also classified into subtypes according to Parchi et al. [3] (i.e., MM(V)1, VV2, MV2K, etc.). Among these, 20 patients showing a mixed subtype were classified based on the dominant histotype according to published criteria [25]. For the biomarker analysis according to the molecular subtype, we merged the patients with definite sCJD with those with a probable diagnosis and a high level of certainty for a given subtype, as previously described [26, 27]. However, we also analyzed the data limited to definite cases to avoid a possible misdiagnosis bias. Further details regarding the classification of patients with probable sCJD are reported in the Supplementary methods (see Additional file 1). gCJD cases were classified in groups based on the combination of PrP^Sc^ type (1, 2 or “i”) and codon 129 *PRNP* genotype in the mutated allele according to Baiardi et al. [4] (i.e., M1, M2C, M2T, etc.).

Regarding the SNAP-25 and Ng prognostic value analyses in CJD, we calculated survival as the time (in months) from LP to death or akinetic mutism. The latter was used in place of time to death exclusively when the revision of medical charts indicated the adoption of life-extending treatments (e.g., enteral/parenteral nutrition, tracheostomy). Five patients were excluded from the survival analyses due to insufficient information on disease duration. Furthermore, we calculated the disease stage in CJD cases as the ratio between disease onset to LP and the overall survival [21, 26].

Within the np-RPD cohort, patients were divided into two main groups depending on whether the neurological syndrome had a degenerative etiology (rp-ND) or not (i.e., RPD due to alternative non-neurodegenerative causes). A third "mixed" group was created to include 14 cases presenting the co-occurrence of two or more central nervous system (CNS) pathologies influencing the clinical phenotype and belonging to both previously cited categories. Further details regarding the clinical-pathological entities included in the np-RPD cohort are reported in the Supplementary methods and shown in Supplementary table 1 (see Additional file 1).

### CSF biomarker analysis

CSF samples were obtained by LP following a standard procedure, centrifuged in case of blood contamination, divided into aliquots, and stored in polypropylene tubes at − 80 °C until analysis. CSF total tau (t-tau) and p-tau were measured by chemiluminescent enzyme immunoassays on the automated Lumipulse G600II platform (Fujirebio), as described [28]. The inter-assay coefficients of variation (CVs) were < 8% for both biomarkers. We used commercially available ELISA kits to measure CSF NfL and 14–3-3 gamma isoform, as described [14, 29]. SNAP-25 concentrations were determined by running the commercially available SNAP-25 Advantage Kit (Quanterix) on the SIMOA SR-X platform (Quanterix). Ng concentrations were assessed with ELISA technology using the Human Neurogranin (Trunc P75) ELISA Kit (EUROIMMUN). The intra-assay and the inter-assay CVs were < 8% and < 15%, respectively, for all four biomarkers. As previously reported, all CSF samples from patients without autopsy examination, classified as probable sCJD or np-RPD, were tested by second-generation prion CSF RT-QuIC [9].

### Statistical analysis

Statistical analyses were performed using GraphPad Prism 9 (Graph-Pad Software) and Stata 14.2 SE (StataCorp). Data were expressed as mean ± standard deviation (SD) or median and interquartile range (IQR) based on the distribution of values. For continuous variables, we variably applied the Mann–Whitney U test, t-test, Kruskal–Wallis test (followed by Dunn-Bonferroni post hoc test), or the one-way analysis of variance (followed by Tukey's post hoc test), depending on the data distribution and the number of groups. All reported *P*-values were adjusted for multiple comparisons, and differences were considered statistically significant at *P* < 0.05. The Chi-square test was adopted for categorical variables. ROC analyses were performed to calculate each biomarker's (and biomarkers ratios) sensitivity, specificity, and diagnostic accuracy with a relative 95% confidence interval (95% CI). We used maximized Youden's index to define the optimal cut-off value for each biomarker. The De Long test was used to compare the areas under the curve between ROC curves. For survival analysis, biomarkers concentration was naturally log-transformed to fulfill the normal distribution. We used the Kaplan–Meier estimate to calculate the cumulative time-dependent probability of death. Univariate and multivariate Cox regression analyses were then performed to assess the association between survival, continuous values or tertiles of each biomarker, and other variables known as prognostic factors in prion disease (age at LP, time from symptoms onset to LP, codon 129 genotype, and clinicopathological subtype) [26, 30]. Survival analyses were performed in the whole CJD cohort and in two separate subgroups, according to clinicopathological subtype as follows: (1) “typical CJD”, including all cases related to the *PRNP* codon 129-M genotype and PrP^Sc^ type 1 combination (namely, sCJD MM(V)1 and gCJD M1); (2) “non-MM(V)1 CJD” including all the other subtypes. The survival analysis results are presented as hazard ratios (HRs) and 95% CIs. The assumption of proportional hazard was assessed by Schoenfeld residuals. Spearman’s correlations were performed to test the possible association between biomarkers concentrations and disease stage.

### Data availability

Anonymized data not published within this article will be made available by request from any qualified investigator.

## Results

### Demographic variables and CSF biomarkers value distribution in the diagnostic groups

**Fig. 1:**
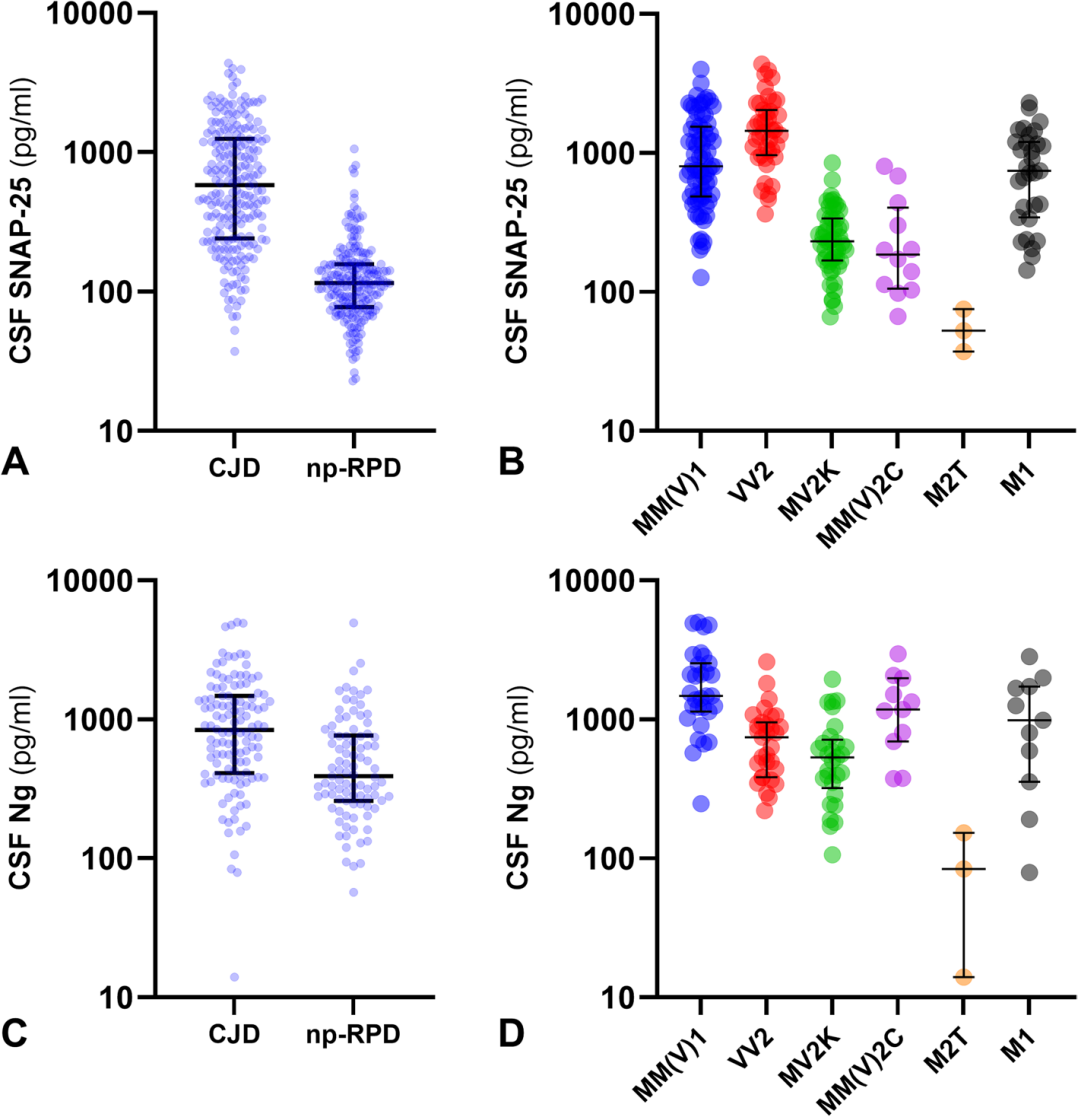
Biomarker levels in the main diagnostic groups and CJD subtypes. CSF SNAP-25 (**A**) and Ng (**C**) levels in Creutzfeldt-Jakob disease (CJD) and non-prion rapidly progressive dementia (np-RPD). CSF SNAP-25 (**B**) and Ng (**D**) levels in sporadic CJD (sCJD) subtypes MM(V)1, VV2, MV2K, and MM(V)2C, and genetic CJD (gCJD) subtypes M2T and M1. Thick lines represent medians and interquartile ranges. CSF SNAP-25 and Ng values are expressed in a logarithmic scale. See the main text for all the *P*-values (Kruskal–Wallis followed by Dunn-Bonferroni post hoc test). Only subgroups comprising at least three cases are shown. Ng, neurogranin; SNAP-25, synaptosomal-associated protein 25

Demographic variables and CSF biomarkers' results in the main diagnostic groups are shown in Table 1 and Fig. 1. Patients with CJD were significantly younger than those suffering from np-RPD (*P* < 0.0001). There was no significant difference between sex distribution across the two diagnostic groups. Results regarding t-tau, NfL, and 14–3-3 were in line with those previously reported [14]. P-tau concentrations were not significantly different between CJD and np-RPD cases. Patients with CJD showed significantly higher CSF SNAP-25 (*P* < 0.0001) and CSF Ng (*P* < 0.0001) levels than those with np-RPD.

**Table 1:** Demographic characteristics of the whole CJD and np-RPD cohorts and distribution of SNAP-25 and Ng levels in the main diagnostic groups

**Diagnostic group**	**Age at LP**	**Females** (%)	**N**	**CSF SNAP-25** (pg/mL)	**N**	**CSF Ng** (pg/mL)
**CJD**	66.5 ± .9.9	48.6	220	582 (240–1250)	122	841 (411–1473)
**sCJD**^**a**^	67.5 ± 9.8	47.0	183	533 (249–1311)	106	868 (471–1430)
• sCJD MM(V)1	69.2 ± 9.5	35.2	71	798 (485–1559)	31	1476 (1139–2527)
• sCJD VV2	68.7 ± 9.8	41.8	43	1438 (962–2034)	31	745 (384–951)
• sCJD MV2K	66.0 ± 9.5	60.3	53	230 (168–338)	29	531 (320–715)
• sCJD MM(V)2C	62.6 ± 8.5	75.0	12	186 (105–403)	11	1179 (695–1975)
• sCJD MM2T	43, 76	50.0	2	85.7, 348.3	2	404, 1751
• sCJD VV1	49, 64	50.0	2	440.5, 512.9	2	1280, 1716
**gCJD**^**b**^	61.9 ± 10.6	54.0	37	662 (230–1173)	16	698 (154–1708)
• gCJD M1	63.3 ± 10.5	54.8	31	744 (344–1200)	11	983 (355–1718)
• gCJD M “i “	49, 68	50.0	2	107.2, 121.7	1	-, 157
• gCJD M2T	45, 68, 49	33.3	3	37.2, 52.5, 75.3	3	14, 84, 153
• gCJD V1	49	100.0	1	334.3	1	1785
**np-RPD**	70.9 ± 11.3	52.1	213	115 (78–157)	93	390 (260–766)
**Non-neurodegenerative**	67.4 ± 11.7	45.9	98	114 (70–162)	43	416 (228–945)
**rp-ND**	73.1 ± 9.9	59.4	101	114 (81–156)	45	354 (273–646)
**Mixed**	74.0 ± 6.9	42.8	14	125 (70–146)	5	787 (355–956)

Age at LP is expressed as mean (SD), while biomarker data are presented as median (IQR)

*Abbreviations*: *CSF* cerebrospinal fluid, *gCJD* genetic Creutzfeldt-Jakob disease, *Ng* neurogranin, *np-RPD* non-prion rapidly progressive dementia, *rp-ND* neurodegenerative np-RPD, *sCJD* sporadic Creutzfeldt-Jakob disease, *SNAP-25* synaptosomal-associated protein 25, *LP* lumbar puncture

^a^Both patients with a definite diagnosis of a specific subtype and patients with a probable diagnosis and a high level of certainty for a given subtype are included

^b^M1 group includes 13 gCJD E200K-129 M, 1 gCJD V203I-129 M and 17 gCJD V210I-129 M; M “i” group includes 2 gCJD E200K-129 M; M2T group includes 3 fatal familial insomnia (FFI) (gCJD D178N-129 M); V1 group includes 1 gCJD D178N-129 V

There was no significant difference in CSF SNAP-25 and Ng concentrations between sCJD and gCJD participants. Within the np-RPD group, CSF SNAP-25 and Ng levels did not vary significantly across the three diagnostic categories.

### Distribution of CSF SNAP25 and Ng according to CJD subtypes

CSF SNAP-25 and Ng distribution in CJD subtypes is shown in Table 1 and Fig. 1. After stratification according to the sCJD subtype, MM(V)1 and VV2 patients showed significantly higher levels of CSF SNAP-25 compared to the other groups (MM(V)1 vs. MV2K, *P* < 0.0001; MM(V)1 vs. MM(V)2C, *P* = 0.0006; VV2 vs. MV2K, *P* < 0.0001; VV2 vs. MM(V)2C, *P* < 0.0001). SNAP-25 levels did not differ significantly between MM(V)1 and VV2 cases. Among the genetic cases, M1 patients showed SNAP-25 levels in the range of MM(V)1 and VV2 groups and significantly higher than MV2K (M1 vs. MV2K, *P* < 0.0001) and MM(V)2C (M1 vs. MM(V)2C, *P* = 0.0276) cases. Finally, M2T patients had the lowest CSF SNAP-25 levels of the whole CJD cohort, significantly lower than MM(V)1 (M2T vs. MM(V)1, *P* = 0.0147), and VV2 (M2T vs. VV2, *P* = 0.0005). All findings previously mentioned remained statistically significant after excluding the probable sCJD cases, except for the comparisons between M1 and MM(V)2C and between MM(V)1 and MM(V)2C.

Regarding the distribution of CSF Ng levels according to sCJD subtypes, sCJD MM(V)1, patients showed the highest values of the whole cohort, with a statistically significant difference when compared to VV2 (MM(V)1 vs. VV2, *P* = 0.0248) and MV2K (MM(V)1 vs. MV2K, *P* < 0.0001) participants. VV2 cases had CSF Ng concentrations in the range of MV2K patients. MM(V)2C and the two VV1 cases showed a CSF Ng concentration in the MM(V)1 group range. M2T cases had the lowest CSF Ng levels of the CJD cohort.

The profiles of the remaining CSF biomarkers stratified by prion disease subtypes are shown in Supplementary table 2 (see Additional file 1).

### Diagnostic performance of CSF biomarkers in the differential diagnosis between CJD and np-RPD

**Fig. 2:**
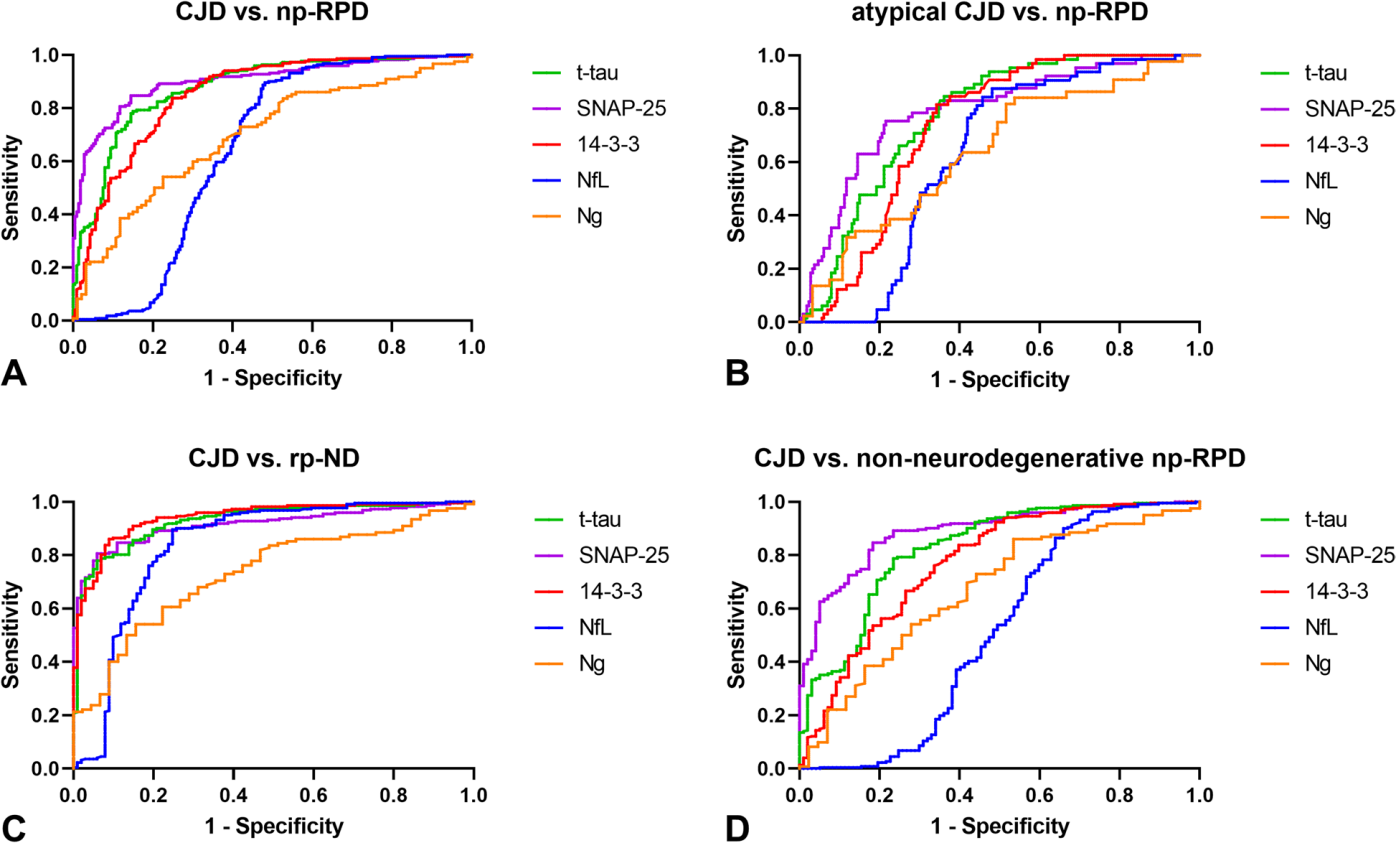
Biomarker diagnostic performance. ROC curves for CSF SNAP-25 (purple), CSF t-tau (green), CSF 14–3-3 (red), CSF NfL (blue), and CSF Ng (orange) in the comparisons between CJD vs. np-RPD (**A**), atypical CJD (MV2K, MM(V)2C, MM2TM and VV1 subtypes of sCJD) vs. np-RPD (**B**), CJD vs. rp-ND (**C**), and CJD vs non-neurodegenerative np-RPD (**D**). CJD, Creutzfeldt-Jakob disease; CSF, cerebrospinal fluid; NfL, neurofilament light chain; Ng, neurogranin; np-RPD, non-prion rapidly progressive dementia; p-tau, phospho-tau181; ROC, receiver operating characteristic; rp-ND, neurodegenerative np-RPD; SNAP-25, synaptosomal-associated protein 25; t-tau, total tau. AUC values for Ng were calculated considering a cohort of 215 patients (122 CJD, 93 np-RPD)

To assess the diagnostic performance of CSF biomarkers, we calculated ROC curves, sensitivity, and specificity for all biomarkers, including their ratios with p-tau. Detailed ROC curve analyses for CSF biomarkers are reported in Table 2 and Fig. 2.

**Table 2:** Diagnostic performance of SNAP-25, Ng, and other surrogate biomarkers

	**CJD vs. np-RPD**				**Atypical CJD**^**a**^** vs. np-RPD**			
	**AUC** **(95% CI)**	**Sensitivity (95%CI)**	**Specificity (95% CI)**	**Cut-off (pg/ml)**	**AUC** **(95% CI)**	**Sensitivity (95%CI)**	**Specificity (95% CI)**	**Cut-off (pg/ml)**
t-tau	0.878 (0.845–0.901)	78.8% (72.9–83.7)	84.9% (79.5–89.1)	1770	0.774 (0.719–0.830)	84.6% (73.9–91.4)	63.8% (57.2–70.0)	993.5
14–3-3	0.853 (0.816–0.889)	83.8% (78.3–88.0)	75.1% (68.9–80.4)	21,750	0.733 (0.676–0.791)	81.5% (70.4–9.1)	65.7% (59.1–71.7)	14,850
NfL	0.649 (0.593–0.705)	89.6% (84.9–92.9)	51.9% (45.2–58.5)	2245	0.626 (0.560–0.691)	87.5% (77.2–93.5)	51.8% (45.1–58.5)	2245
SNAP-25	0.902 (0.873–0.931)	84.7% (79.3–88.8)	85.4% (80.0–89.5)	198.7	0.792 (0.729–0.854)	75.3% (63.6–84.2)	78.4% (72.4–83.4)	163.8
Ng^b^	0.697 (0.626–0.767)	54.1% (45.2–62.6)	77.4% (67.9–84.7)	793.5	0.643 (0.545–0.741)	84.0% (70.6–92.0)	46.2% (36.4–56.3)	350.0
t-tau/p-tau	0.884 (0.851–0.916)	92.2% (87.8–95.0)	70.4% (63.8–76.2)	17.80	0.778 (0.725–0.830)	98.4% (91.5–99.9)	63.6% (56.8–69.8)	11.90
SNAP-25/p-tau	0.924 (0.900–0.948)	92.2% (87.8–95.0)	79.6% (73.6–84.5)	2.950	0.812 (0.760–0.864)	77.8% (66.0–86.2)	79.6% (73.6–84.5)	2.950
	**CJD vs. rp-ND**				**CJD vs. non-neurodegenerative np-RPD**			
	**AUC** **(95% CI)**	**Sensitivity (95%CI)**	**Specificity (95% CI)**	**Cut-off (pg/ml)**	**AUC** **(95% CI)**	**Sensitivity (95%CI)**	**Specificity (95% CI)**	**Cut-off (pg/ml)**
t-tau	0.928 (0.899–0.957)	77.9% (72.0–82.9)	94.0% (87.6–97.2)	1808	0.826 (0.775–0.877)	78.8% (72.9–83.7)	76.5% (67.2–83.8)	1768
14–3-3	0.942 (0.916–0.967)	86.0% (80.8–89.9)	91.0% (83.9–95.2)	18,850	0.771 (0.712–0.831)	83.8% (78.4–88.0)	60.2% (50.3–69.3)	21,750
NfL	0.839 (0.782–0.896)	89.6% (84.9–92.9)	75.2% (66.0–82.6)	2245	0.512 (0.429–0.595)	90.0% (85.4–93.3)	34.0% (25.4–43.9)	15,450
SNAP-25	0.916 (0.886–0.946)	80.6% (74.9–85.3)	94.0% (87.6–97.2)	214.2	0.887 (0.850–0.925)	84.7% (79.4–88.8)	81.6% (72.8–88.0)	195.3
Ng^b^	0.733 (0.652–0.813)	54.1% (45.2–62.6)	84.4% (71.2–92.2)	787.0	0.669 (0.573–0.765)	86.0% (78.8–91.1)	46.5% (32.5–61.0)	339.0
t-tau/p-tau	0.966 (0.943–0.989)	98.6% (96.0–99.6)	88.0% (80.1–93.0)	11.65	0.794 (0.736–0.851)	63.7% (57.1–69.8)	82.6% (73.6–89.0)	51.40
SNAP-25/p-tau	0.971 (0.954–0.987)	92.2% (87.9–95.0)	93% (86.2–96.6)	2.950	0.869 (0.829–0.910)	78.4% (72.5–83.3)	83.7% (74.8–89.8)	4.950

*Abbreviations*: *AUC* area under the curve, *CI* confidence interval, *CSF* cerebrospinal fluid, *NfL* neurofilament light chain, *Ng* neurogranin, *np-RPD* non-prion rapidly progressive dementia, *p-tau* phospho-tau181, *rp-ND* neurodegenerative np-RPD, *SNAP-25* synaptosomal-associated protein 25; t-tau, total tau

^a^sCJD subtypes MV2K, MM(V)2C, MM2T, and VV1 are included

^b^AUC values for Ng were calculated considering a cohort of 215 patients (122 CJD, 93 np-RPD)

In ROC curves analysis, CSF SNAP-25 and Ng yielded a diagnostic accuracy of respectively 90% (area under the curve [AUC] 0.902 [0.873–0.931]) and 69% (AUC 0.697 [0.626–0.767]) in discriminating between CJD and np-RPD. The diagnostic performance of CSF SNAP-25 was better than that of t-tau (AUC 0.878 [0.845–0.901]) (SNAP-25 vs. t-tau, *P* = 0.0179), 14–3-3 (AUC 0.853 [0.816–0.889]) (SNAP-25 vs. 14–3-3, *P* = 0.0005), and NfL (AUC 0.649 [0.593–0.705]) (SNAP-25 vs. NfL, *P* < 0.0001). The diagnostic accuracy of CSF Ng slightly exceeded that of NfL (Ng vs. NfL, *P* = 0.0209) but was inferior to that of SNAP-25 (Ng vs. SNAP-25, *P* < 0.0001), t-tau (Ng vs. t-tau, *P* = 0.0002), and 14–3-3 (Ng vs. 14–3-3, *P* = 0.0153). The combined use of p-tau increased the diagnostic accuracy of both synaptic biomarkers, with the CSF SNAP-25/p-tau ratio performing slightly better than the t-tau/p-tau ratio (AUC 0.924 [0.900–0.948]) (SNAP-25/p-tau vs. t-tau/p-tau, *P* = 0.0025) (Table 2).

In the differential diagnosis between the "atypical," slowly progressive sCJD subtypes (MV2K, MM(V)2C, MM2T, and VV1) and np-RPD, CSF SNAP-25 diagnostic accuracy (AUC 0.792 [0.729–0.854]) was in the range of that of t-tau, t-tau/p-tau ratio, and SNAP-25/p-tau ratio. CSF SNAP-25 and t-tau outperformed CSF Ng in this sub-analysis (SNAP-25 vs. Ng, *P* < 0.0001; t-tau vs. Ng, *P* = 0.0169).

When we restricted the analysis to the most common sCJD subtypes (MM(V)1 and VV2), CSF SNAP-25 yielded an extremely high diagnostic accuracy (AUC 0.982 [0.971–0.994]). In this sub-analysis, the correction of SNAP-25 values with p-tau did not significantly impact the diagnostic performance of the biomarker (AUC 0.988 [0.979–0.996]). In the same comparison, CSF Ng diagnostic value (as that of the other biomarkers) improved as well (AUC 0.809 [0.727–0.891]), although its diagnostic power remained way lower than that of SNAP-25, t-tau, and 14–3-3.

### Diagnostic performance of CSF biomarkers in the differential diagnosis between CJD and non-neurodegenerative np-RPD

When we assessed the biomarkers' diagnostic performance in distinguishing between patients with CJD and those with non-neurodegenerative np-RPD, CSF SNAP-25 outperformed all the other single and p-tau-adjusted biomarkers (AUC 0.887 [0.850–0.925]) (SNAP-25 vs. t-tau, *P* = 0.0003; SNAP-25 vs. t-tau/p-tau, *P* = 0.0002; SNAP-25 vs 14–3-3, *P* < 0.0001; SNAP-25 vs. NfL, *P* < 0.0001) (Table 2). CSF Ng diagnostic value (AUC 0.669 [0.573–0.765]) was significantly lower than that of SNAP-25 (SNAP-25 vs. Ng, *P* = 0.0003). In this analysis, p-tau-corrected biomarkers yielded a lower diagnostic accuracy than the single correspondent biomarkers (Table 2).

### Diagnostic performance of CSF biomarkers in the differential diagnosis between CJD and rp-ND

**Fig. 3:**
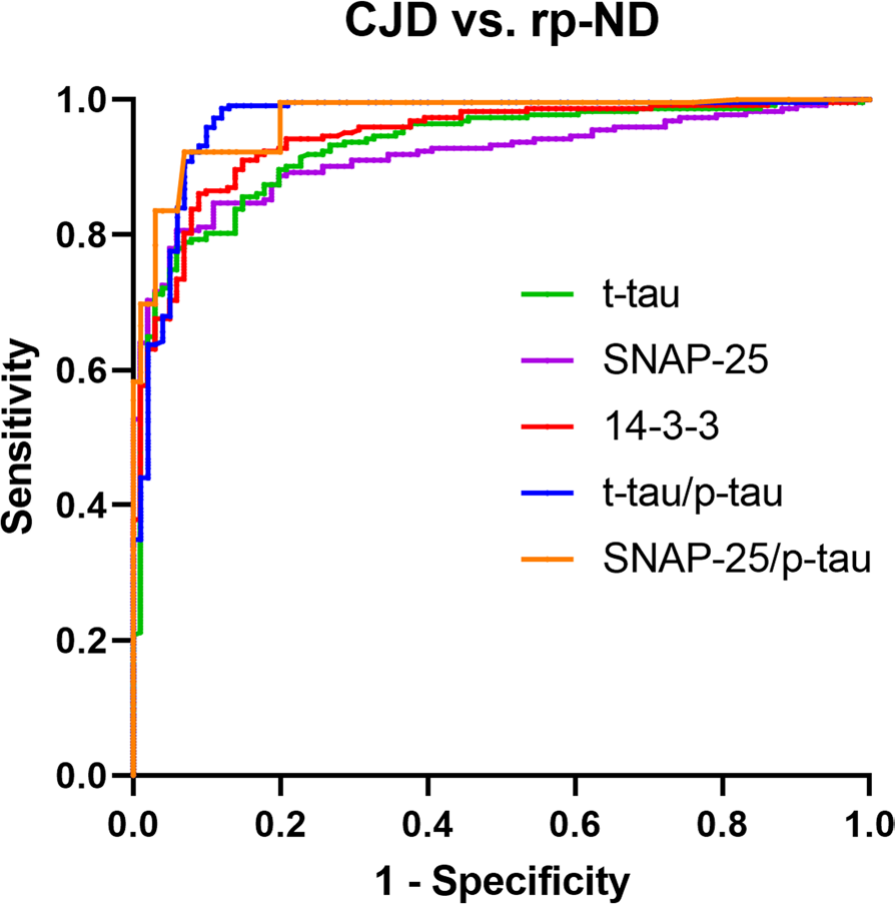
Biomarker diagnostic performance in the CJD vs rp-ND setting. ROC curves for CSF SNAP-25 (purple), CSF t-tau (green), CSF 14–3-3 (red), t-tau/p-tau (blue), and SNAP-25/p-tau (orange) in the comparisons between CJD vs. rp-ND. CJD, Creutzfeldt-Jakob disease; CSF, cerebrospinal fluid; p-tau, phospho-tau181; ROC, receiver operating characteristic; rp-ND, neurodegenerative non-prion rapidly progressive dementia; SNAP-25, synaptosomal-associated protein 25; t-tau, total tau

When analyzing the biomarkers diagnostic accuracy in discriminating between patients with CJD and those with an rp-ND, CSF SNAP-25 (AUC 0.916 [0.886–0.946]) was outperformed by 14–3-3 (AUC 0.942 [0.916–0.967]) (SNAP-25 vs. 14–3-3, *P* = 0.0203) (Table 2). SNAP-25 diagnostic value was in the range of that of t-tau (AUC 0.928 [0.899–0.957]). When considering the p-tau corrected values, the CSF SNAP-25/p-tau, and t-tau/p-tau ratios yielded the highest diagnostic accuracy among the single and the p-tau-adjusted biomarkers (AUC 0.971 [0.954–0.987] and AUC 0.966 [0.943–0.989], respectively) (Table 2). CSF Ng performed worse than all the other biomarkers (Ng vs. t-tau, *P* < 0.0001; Ng vs. 14–3-3, *P* < 0.0001; Ng vs. SNAP-25, *P* < 0.0001), except for NfL (AUC 0.839 [0.782–0.896]). In this analysis, all p-tau-corrected biomarkers showed better diagnostic accuracy than the corresponding single biomarkers (Table 2). Detailed ROC curves of p-tau adjusted and single best-performing biomarkers in this sub-analysis are shown in Fig. 3.

### Prognostic value and association with disease stage of CSF SNAP-25 and Ng in CJD

When considering the whole CJD cohort, CSF SNAP-25 was significantly associated with survival (HR 1.71 [1.48–1.99], *P* < 0.001), even after accounting for covariates known as prognostic factors in prion disease as codon 129 genotype, age at LP and time from onset to LP. When stratifying for the clinicopathological subtype, CSF SNAP-25 levels correlated with survival in both “typical” MM(V)1 CJD (HR 1.36 [1.03–1.80], *P* = 0.029) and “non-MM(V)1 CJD” (HR 1.69 [1.38–2.06], *P* < 0.001) groups. In the multivariate Cox regression, CSF SNAP-25 were significantly associated with survival in both the whole prion cohort (HR 1.71 [1.40–2.09], *P* < 0.001), and “typical CJD” (HR 1.52 [1.15–2.02], *P* = 0.003), but not in “non-MM(V)1 CJD”.

Conversely, we found no significant associations between CSF Ng and survival when considering the whole CJD cohort and the “non-MM(V)1 CJD” subtypes. CSF Ng levels significantly correlated with survival in “typical CJD” in both univariate and multivariate Cox regression (HR 1.50 [1.04–2.16], *P* = 0.027; and HR 1.81 [1.21–2.93], *P* = 0.015; respectively).

Within the whole CJD cohort, CSF t-tau, 14–3-3, and NfL were significantly associated with survival in both univariate (HR 1.73 [1.49–2.02], *P* < 0.001; HR 1.72 [1.46–2.02], *P* < 0.001; and HR 1.27 [1.09–1.49], *P* = 0.002, respectively) and multivariate Cox regression analyses, yielding a prognostic performance in the range of that of SNAP-25 (in case of t-tau and 14–3-3) and Ng (in case of NfL).

**Fig. 4:**
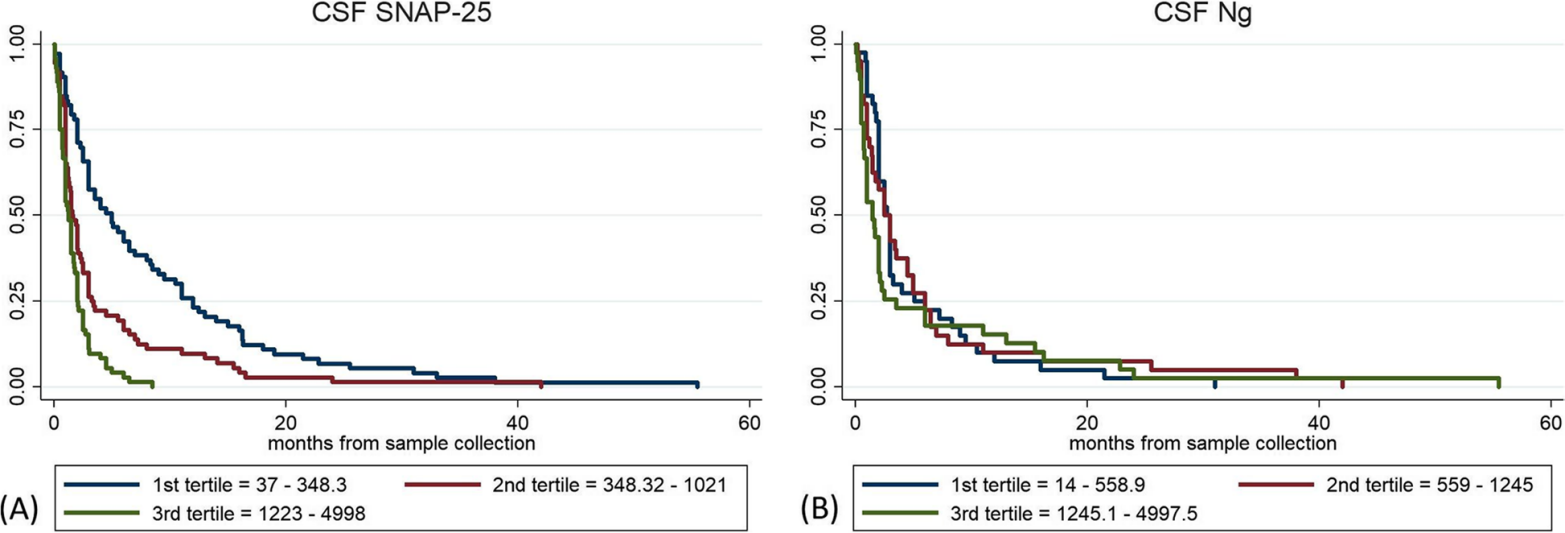
Biomarker prognostic performance. Prognostic value of CSF SNAP-25 (**A**) and Ng (**B**). Survival curves in patients of the whole CJD cohort according to the values of CSF synaptic biomarkers. CSF, cerebrospinal fluid, Ng, neurogranin; SNAP-25, synaptosomal-associated protein 25

Detailed data regarding the association between CSF SNAP-25 and Ng levels and survival are shown in Table 3. Survival curves are shown in Fig. 4. Survival analysis results for t-tau, 14–3-3, and NfL are reported in Supplementary table 3 and shown in Supplementary Fig. 1 (see Additional file 1).

**Table 3:** Associations of SNAP-25 and Ng CSF levels with survival time in the whole CJD cohort and after stratification according to the disease subtype

Diagnostic group and biomarker		Survival time	Univariate Cox regression		Multivariate Cox regression^a^	
		**Median ± IQR (months)**	**HR (95% CI)**	***P* value**	**HR (95% CI)**	***P* value**
**Whole CJD cohort**						
**SNAP-25 (*N* = 215)**	Continuous value	5.0 (2.8–12.0)	**1.71 (1.48–1.99)**	** < .001**	**1.71 (1.40–2.09)**	** < .001**
	Low tertile	12.0 (6.0–17.5)	Ref	Ref	Ref	Ref
	Mid tertile	4.0 (2.5–9.0)	**1.87 (1.34–2.61)**	** < .001**	**1.71 (1.19–2.44)**	**.003**
	High tertile	4.0 (2.5–5.5)	**3.42 (2.38–4.90)**	** < .001**	**3.42 (2.15–5.45)**	** < .001**
**Ng (*N* = 119)**	Continuous value	6.0 (3.6–13.0)	1.19 (0.99–1.45)	.065	1.04 (0.82–1.33)	.720
	Low tertile	7.5 (5.4–14.0)	Ref	Ref	Ref	Ref
	Mid tertile	6.0 (3.5–11.8)	0.99 (0.63–1.54)	.971	0.80 (0.49–1.32)	.396
	High tertile	4.1 (2.6–12.0)	1.23 (0.78–1.92)	.374	0.93 (0.54–1.60)	.808
**Typical CJD**^**b**^						
**SNAP-25 (*N* = 100)**	Continuous value	2.8 (2.1–4.0)	**1.36 (1.03–1.80)**	**.029**	**1.52 (1.15–2.02)**	**.003**
	Low tertile	3.5 (2.4–4.1)	Ref	Ref	Ref	Ref
	Mid tertile	2.5 (2.0–3.4)	0.94 (0.53–1.65)	.831	1.03 (0.56–1.77)	.991
	High tertile	2.5 (2.1–3.3)	1.35 (0.76–2.39)	.302	1.65 (0.91–2.96)	.093
**Ng (*N* = 41)**	Continuous value	3.0 (2.0–4.0)	**1.50 (1.04–2.16)**	**.027**	**1.81 (1.21–2.93)**	**.015**
	Low tertile	2.4 (1.8–4.5)	Ref	Ref	Ref	Ref
	Mid tertile	3.0 (2.1–3.8)	2.48 (0.70–8.76)	.157	**7.90 (1.28–48.71)**	**.026**
	High tertile	3.0 (2.5–3.9)	**3.59 (1.01–12.67)**	**.047**	**10.34 (1.71–62.34)**	**.011**
**non-MM(V)1 CJD**^**c**^						
**SNAP-25 (*N* = 115)**	Continuous value	11.0 (6.0–16.0)	**1.69 (1.38–2.06)**	** < .001**	1.01 (0.73–1.40)	.937
	Low tertile	15.0 (11.0–24.0)	Ref	Ref	Ref	Ref
	Mid tertile	14.8 (10.8–19.7)	1.39 (0.88–2.19)	.147	1.03 (0.58–1.83)	.914
	High tertile	5.5 (4.7–6.5)	**4.68 (2.81–7.78)**	** < .001**	0.91 (0.36–2.29)	.844
**Ng (*N* = 78)**	Continuous value	9.5 (6.0–16.0)	0.93 (0.76–1.15)	.547	0.81 (0.60–1.09)	.169
	Low tertile	9.0 (6.3–15.0)	Ref	Ref	Ref	Ref
	Mid tertile	9.0 (5.9–15.5)	0.69 (0.41–1.15)	.164	0.58 (0.33–1.01)	.058
	High tertile	12.0 (6.0–23.8)	**0.50 (0.26–0.94)**	**.034**	**0.45 (0.22–0.91)**	**.027**

Bold values indicate statistically significant hazard ratios

*Abbreviations*: *CI* confidence interval, *CSF* cerebrospinal fluid, *gCJD* genetic Creutzfeldt-Jakob disease, *HR* hazard ratio, *IQR* interquartile range, *Ng* neurogranin, *Ref* reference, *sCJD* sporadic Creutzfeldt-Jakob disease, *SNAP-25* synaptosomal-associated protein 25

^a^All multivariate Cox regression analyses included codon 129 genotype, age at LP and time from onset to sample collection as covariates

^b^Includes sCJD MM(V)1 and gCJD M1

^c^Includes sCJD VV2, sCJD MV2K, sCJD MM(V)2C, sCJD MM2T, sCJD VV1, gCJD M “i”, gCJD M2T, and gCJD V1

Regarding the possible correlation between CSF biomarkers levels and disease stage within the whole CJD cohort, higher SNAP-25 was weakly associated with later disease stage (SNAP-25: *r* = 0.2487, *P* = 0.0003), while Ng showed no significant association (Ng: *r* = 0.1213, *P* = 0.1927). The disease stage did not correlate with biomarker values across CJD subtypes, apart from SNAP-25 in MV2K (*r* = 0,3981, *P* = 0.0046). Data regarding the association between biomarkers levels and disease stages are shown in Supplementary Fig. 2.

## Discussion

Synaptic proteins released in body fluids due to neurodegeneration have attracted increasing attention as promising candidates for diagnostic markers in neurodegenerative diseases. Indeed, synapses are an early target of PrP^Sc^ deposition, and synaptic loss is an early sign of neurodegeneration in prion disease [16–20]. In recent studies, CSF SNAP-25 and Ng levels yielded a high diagnostic accuracy in distinguishing CJD from other neurodegenerative disorders [21–24]; however, no studies have ever evaluated their diagnostic value in the clinical setting (i.e., against other rapidly progressive neurological syndromes) and the influence of the CJD subtype on biomarker levels.

To assess their diagnostic performance in the clinical routine of a prion disease referral center, we measured CSF SNAP-25 and Ng concentrations in a large RPD cohort comprising both CJD and np-RPD cases. Our analysis confirmed the marked increase of SNAP-25 and Ng CSF levels in patients with CJD compared to other neurological diseases, reflecting the extensive synaptic damage [21, 22, 31]. As the most relevant findings, we showed that the diagnostic value of CSF SNAP-25 outperformed all the other established surrogate biomarkers, including 14–3-3, NfL, and, most notably, t-tau, the latter being considered by many the best-performing surrogate markers in support of the clinical diagnosis of CJD [14].

SNAP-25 diagnostic advantage over t-tau may be partially explained by the increase of CSF levels in the early stages of prion disease, as opposed to those of t-tau, which appear to rise progressively over the disease course [21, 32]. Notably, SNAP-25 maintained the high diagnostic power even for the rare and atypical sCJD subtypes (MV2K, MM2C, MM2T, and VV1) often showing low t-tau and 14–3-3 CSF levels [29], thus making it a valuable biomarker for the whole CJD spectrum. In contrast to SNAP-25, Ng discriminatory power in distinguishing CJD from np-RPD cases was substantially lower than that of t-tau and 14–3-3 and only slightly higher than that of NfL.

Two major clinical scenarios can exemplify the differential diagnosis of CJD in clinical practice: CJD versus np-RPD and CJD versus rp-ND. When comparing diagnostic accuracies in distinguishing CJD from patients with non-neurodegenerative np-RPD (i.e., primarily inflammatory-related conditions and subacute dementias), CSF SNAP-25 discriminatory power exceeded that of t-tau (and the t-tau/p-tau ratio), which is currently considered the gold standard screening test in this specific clinical scenario [11, 15]. The relative preservation of synaptic integrity reported in the acute phase of antibody-mediated encephalitis [33], a common cause of non-neurodegenerative RPD, may, at least partially, justify this diagnostic superiority. In this clinical context, the SNAP-25/p-tau ratio diagnostic accuracy was not significantly different from that of SNAP-25 alone.

The differential diagnosis between CJD and rp-ND, mostly rapidly progressive Alzheimer's disease (AD), is a common clinical scenario in which t-tau yields a discrete number of false positive results. CSF SNAP-25 and t-tau diagnostic power fell slightly behind 14–3-3, currently considered the most helpful assay in this clinical context [11, 15, 34]. Notably, in this analysis, all p-tau-corrected biomarkers showed better diagnostic accuracy than the corresponding single biomarkers. This is likely due to the inclusion in the rp-ND cohort of many patients with significant AD pathology, thus likely exhibiting high CSF p-tau levels [35]. More specifically, when adjusted for p-tau values, both SNAP-25/p-tau and t-tau/p-tau ratios showed a diagnostic performance in the range of 14–3-3. CSF Ng yielded the lowest diagnostic accuracy among the single and the p-tau-adjusted biomarkers. Our results differ from those of a previous study, which reported a high Ng discriminatory power between CJD and AD (AUC 0.85 [0.78–0.92]) [22]. This is most likely due to the inclusion in our cohort of a relatively large number of patients with CJD subtypes showing only a modest increase in CSF Ng levels (i.e., VV2 and MV2K). Moreover, while previous studies mostly comprised "typical" (i.e., non-rapidly progressive) ND as the control group, we only included rp-ND, likely exhibiting higher levels of surrogate neurodegeneration markers, including Ng.

Our results indicate that SNAP-25 is a viable alternative diagnostic marker to 14–3-3 and t-tau for RPDs. Although it is unlikely to provide a significant clinical advantage over t-tau in discriminating between CJD and rp-ND overall, the clinical impact of SNAP-25 could be relevant when the differential diagnosis with non-neurodegenerative np-RPD is an issue. The SNAP-25/p-tau ratio provides no additional diagnostic value compared to SNAP-25 alone, except in the differential diagnosis with rp-ND.

Regarding the synaptic biomarkers distributions across CJD subtypes, the mean CSF SNAP-25 levels were higher in MM(V)1 and VV2 than in MV2K, MM2C, MM2T, and VV1. Patients with gCJD M2T showed the lowest levels of the whole CJD cohort. The distribution pattern of mean CSF SNAP-25 levels among CJD subtypes was substantially similar to that previously reported for CSF t-tau, CSF 14–3-3, and CSF α-synuclein [15, 27]. In contrast, CSF Ng levels were significantly higher in MM(V)1 than in VV2 and MV2K. High values were also reported in the few tested MM(V)2C and VV1 cases. Eventually, as previously shown, CSF NfL values distribution followed a divergent pattern, with the highest concentrations reported in VV2 and, in descending order, in MV2K and MM(V)1 [29]. Numerous pieces of evidence show that CSF levels of surrogate neurodegeneration biomarkers reflect, at least partially, the entity of the brain damage in a certain amount of time. However, they also recapitulate the prevalent regional and subcellular pathology of a given disorder, depending on the cerebral areas in which they are expressed and the subcellular compartment in which they are preferentially localized. In this regard, biomarkers can be classified into "generic neuroaxonal" (e.g., t-tau), "myelinated axonal" (e.g., NfL), and "synaptic" (e.g., α-synuclein). While SNAP-25 likely acts as a traditional synaptic biomarker, Ng may be a marker of both synaptic and generic neuroaxonal damage [22, 27, 36]. Our current and previous results suggest that in CJD, both generic neuroaxonal (t-tau) and some synaptic (at least, α-synuclein and SNAP-25) biomarkers reflect the extent of neuronal (i.e., grey matter) degeneration and the speed at which it develops. In contrast, myelinated axonal biomarkers (NfL) levels correlate with the presence and the degree of subcortical axonal (i.e., white matter) pathology [27, 29].

Interestingly, CSF Ng levels distribution across CJD subtypes slightly differs from those of the other synaptic and neuroaxonal biomarkers, especially regarding the relatively low concentrations observed in VV2. Ng is a neuronal protein, mainly expressed in the cerebral cortex and hippocampus and, to a lesser extent, in the amygdala, caudate, and putamen [37]. Based on such evidence, in line with previous observations [22], we speculate that Ng may act as a "topographic" marker of cortical and hippocampal pathology, not only in AD [37] but also CJD, as demonstrated by the high CSF levels observed in sCJD subtypes with prominent cortical pathology (MM(V)1, MM(V)2C and VV1) and the unexpectedly low concentrations reported in VV2 (in whom subcortical and cerebellar pathology is prevailing) [3]. Though further studies are needed, this peculiar behavior makes it a promising biomarker for the in vivo identification of rare and atypical sCJD subtypes with prominent cortical pathology (i.e., MM(V)2C and VV1).

In the present study, in line with recently proposed prognostic models [38, 39], we carried out the survival analyses also considering clinical variables known to have a prognostic role in prion diseases such as age at LP, *PRNP* codon 129 genotype, and time between symptoms onset and LP. Consistent with previous observations [21], we found a significant association between CSF levels of SNAP-25 at LP and survival, even after accounting for covariates such as codon 129 genotype, age at LP, and time between symptoms onset and LP. Notably, this correlation remained significant even when stratifying the analysis into two subgroups based on the clinicopathological subtype. Conversely, CSF Ng concentrations were significantly associated with survival only in the most rapidly progressive CJD subtypes (sCJD MM(V)1 and gCJD M1) but neither in the whole prion cohort nor in slower-progressive CJD subtypes. These results, apparently at odds with previous studies prospecting some prognostic power for Ng in CJD [22], are likely due to the inclusion in our cohort of a relatively large number of patients with CJD subtypes (i.e., MM(V)2C) exhibiting both prolonged survival and extremely high CSF Ng levels. Notably, in non-MM(V)1 group, Ng behaved as a protective factor (with statistical significance in the third tertiles) probably because Ng levels are higher in the “cortical” sCJD MM(V)2C subtype than in the “subcortical” and faster-progressing sCJDVV2 (mean disease duration 18 vs. 6.5 months).

Regarding the strength of the association between biomarkers levels and survival in the whole CJD cohort, CSF SNAP-25 yielded an HR similar to CSF t-tau, CSF 14–3-3 ones (data not shown), and the one we previously reported for CSF α-synuclein [27]. In contrast, Ng HR (though not statistically significant) was in the range of the NfL one (data not shown). This may be due to the biological peculiarity of the two biomarkers mentioned above. Indeed, while CSF levels of SNAP-25, 14–3-3, t-tau, and α-synuclein reflect the neuronal loss rate in the entire CNS's gray matter, those of NfL and Ng predominantly reflect the neurodegeneration occurring in specific brain areas (subcortical white matter and cortex, respectively).

Regarding the possible correlation between biomarkers levels and clinical variables, we found a weak association between SNAP-25 concentrations and disease stages in the whole cohort. SNAP-25 concentrations did not vary significantly between stages in each sCJD subtype but the MV2K. These results suggest that SNAP-25 levels increase early and remain stable during the disease course in the most common subtypes (i.e., MM(V)1 and VV2), as previously reported [21]. In the case of sCJD MV2K, we speculate that SNAP-25 concentrations increase progressively over the disease course due to the slower neurodegenerative process. This may also prove true for the other long-survival subtypes (e.g., MM(V)2C), for which, however, it is difficult to draw firm conclusions due to the small sample size in our cohort. CSF Ng showed a similar dynamic, as we found no association between biomarker concentrations and disease stage neither in the whole CJD cohort nor in each clinicopathological subtype, in line with previous studies [22]. This observation supports their potential role as early diagnostic and prognostic biomarkers in CJD.

## Limitations

We are aware that the low number of neuropathologically-verified np-RPD cases is a limitation of this study. Similarly, the classification of participants with probable CJD into a specific clinicopathological subtype could have been mistaken due to the lack of a definite diagnosis. However, we are confident that through the thorough screening of medical records, codon 129 genotyping, and the second-generation prion RT-QuIC, we have effectively tackled the risks of patient misdiagnosis and misclassification. We recognize that some of the biomarkers used for sCJD subtype determination (e.g., t-tau) in the cases lacking neuropathology share parts of their pathophysiological background with SNAP-25 or Ng and that this may cause a verification bias when the biomarker levels are compared between (clinically determined) subtypes. As an additional limitation, the fact we used akinetic mutism in place of time to death when life-extending treatments were adopted might have introduced a bias in survival calculation, given that we did not use the same variable for all patients. Unfortunately, the information on the onset of akinetic mutism was unavailable for some patients. However, we thought that eliminating the significant effect of life-extending treatments would be more accurate than non-considering this variable, which would also introduce a bias in the calculation of disease duration among patients. We also know that small sample sizes are an additional study limitation, especially in subanalyses concerning rare sCJD subtypes (MM(V)2C, MM2T, and VV1). Finally, this study's retrospective and unicentric nature could limit the generalizability of the results. As the main strength, in this study, we assessed the diagnostic value of CSF SNAP-25 and Ng in a large cohort of patients presenting with a rapidly progressive neurological syndrome, thus reflecting the clinical scenario in which these biomarkers are expected to be employed.

## Conclusions

In conclusion, our results suggest that CSF SNAP-25 is a viable alternative to established diagnostic CSF surrogate biomarkers such as t-tau, 14–3-3, and the t-tau/p-tau ratio in discriminating CJD among RPD cases, especially from those of non-neurodegenerative etiology. When differential diagnosis with rp-ND is an issue, the biomarkers' accuracy can be optimized by calculating the SNAP-25/p-tau ratio. In contrast to SNAP-25, CSF Ng diagnostic performance is significantly worse than that of CSF t-tau and CSF 14–3-3. Moreover, our study suggests that CSF SNAP-25 and, to a lesser extent, CSF Ng can predict survival in CJD, with the former yielding a predictive performance comparable to that of CSF t-tau, 14–3-3, and α-synuclein. Finally, since CSF levels of synaptic biomarkers seem to recapitulate the prevalent regional and sub-cellular pathology of prion diseases, they could prospectively serve as valuable tools for patient stratification in clinical trials and patient-tailored medical management.

### Supplementary Information

**Additional file 1: Supplementary material. Supplementary methods**, probable sCJD and np-RPD patients’ classification. **Supplementary table 1**, np-RPD cohort diagnostic categories. **Supplementary table 2**, distribution of biomarkers levels in the subgroups of the CJD cohort. **Supplementary table 3**, associations of t-tau, 14-3-3, and NfL CSF levels with survival time in the whole CJD cohort and after stratification according to the disease subtype. **Supplementary figure 1**, t-tau, 14-3-3, and NfL prognostic performance. **Supplementary figure 2**, Correlation analysis between SNAP-25 or Ng levels and disease stages.

## Data Availability

The datasets used and analyzed during the current study are available from the corresponding author upon reasonable request.

## Abbreviations

AD: Alzheimer’s disease
CI: Confidence interval
CJD: Creutzfeldt-Jakob disease
CNS: Central nervous system
CSF: Cerebrospinal fluid
CV: Coefficient of variation
DW-MRI: Diffusion weighted- magnetic resonance imaging
gCJD: Genetic Creutzfeldt-Jakob disease
HR: Hazard ratio
LP: Lumbar puncture
NfL: Neurofilament light chain
Ng: Neurogranin
np-RPD: Non-prion rapidly progressive dementia
PrP: Prion protein
PrP^sc^: Misfolded prion protein
*PRNP*: Prion protein gene
p-tau: Phospho-tau181
ROC: Receiver operating characteristics
RT-QuIC: Real-time quaking-induced conversion
rp-ND: Neurodegenerative rp-RPD
sCJD: Sporadic Creutzfeldt-Jakob disease
SNAP-25: Synaptosomal-associated protein 25
t-tau: Total-tau
