# Supplementary material for: Diagnostic and prognostic value of cerebrospinal fluid SNAP-25 and neurogranin in Creutzfeldt-Jakob disease in a clinical setting cohort of rapidly progressive dementias
Source: Alzheimers Res Ther. 2023 Sep 8;15:150. doi: 10.1186/s13195-023-01300-y (PMC10485978; doi:10.1186/s13195-023-01300-y)
Supplement: Supplementary file 1 — Additional file 1: Supplementary material. Supplementary methods, probable sCJD and np-RPD patients’ classification. Supplementary table 1, np-RPD cohort diagnostic categories. Supplementary table 2, distribution of biomarkers levels in the subgroups of the CJD cohort. Supplementary table 3, associations of t-tau, 14-3-3, and NfL CSF levels with survival time in the whole CJD cohort and after stratification according to the disease subtype. Supplementary figure 1, t-tau, 14-3-3, and NfL prognostic performance. Supplementary figure 2, Correlation analysis between SNAP-25 or Ng levels and disease stages. [file 13195_2023_1300_MOESM1_ESM.docx]

**SUPPLEMENTARY MATERIAL**

**Supplementary methods**

**Probable sCJD patients’ classification**

The classification of probable sCJD subtypes was determined by the consensus of two consultant neurologists (SB and PP) after reviewing genetic testing results (codon 129 genotype), clinical features, CSF biomarkers, and brain MRI as previously described (1,2).

Probable sCJD VV2 cases were homozygotes VV at codon 129, showed prominent rapidly progressive early ataxia and a disease duration < 12 months, were positive at prion RT-QuIC (3), and had at least 2 of the following: positive 14-3-3 and/or t-tau levels > 1250 pg/ml, prominent striatum and/or thalamic involvement at brain MRI in the early phase of the disease (3–7). They showed averagely high values of CSF 14-3-3 and t-tau [109000 AU (74900-171000) and 9195 pg/ml (5001-13300), respectively], as described (3,8,9). Patients (129-VV) with clinical features highly suggestive of the VV1 subtype (i.e., age at onset ≤36 years, disease duration ≥17 months, and predominant cortical symptoms/signs without ataxia (7)) were excluded and classified accordingly.

Probable sCJD MV2K were heterozygotes MV at codon 129, presented with prominent ataxia and/or cognitive decline at onset, had a disease duration > 8 months (4,6), were positive at prion RT-QuIC (3) and brain MRI (DWI/FLAIR sequences) (5). Furthermore, probable MV2K patients showed relatively low CSF 14-3-3 [23100 AU (16500-33400)] and t-tau levels [1688 pg/ml (1267-2397)], respectively) as previously described (3,8,9). Patients (129-MV) exhibiting clinical features highly suggestive of the MV1 subtype (disease duration < 8 months and presenting with a multisystemic neurological syndrome (10)) were excluded and classified accordingly as previously reported (1,2).

Probable sCJD MM2C were homozygotes MM at codon 129, showed a prominent cognitive decline at onset, had a disease duration > 8 months (4,6), and were positive at prion RT-QuIC (3) and at brain MRI (DWI/FLAIR sequences) (5). Moreover, probable MM2C showed low levels of CSF 14-3-3 [21250 AU (11600-44050)] and t-tau [1382 pg/ml (1011-4117)], as described (3,8,9). Patients (129 MM) with disease duration < 6 months and presenting with a multisystemic neurological syndrome were classified as MM1.

**np-RPD patients’ classification**

Patients belonging to this group presented with RPD and tested negative by prion RT-QuIC. They were divided into seven categories on an etiologic basis: inflammatory (e.g., immune-mediated or infectious encephalitis), epilepsy (e.g., recurrent seizures, status epilepticus), toxic-metabolic (e.g., Wernicke–Korsakoff syndrome, hepatic encephalopathy, uremia), neoplastic (primitive or secondary CNS malignancies), vascular (e.g., recurrent or progressive strokes, vascular dementia), psychiatric (e.g., affective disorders, delirium), and degenerative (rp-ND). A “mixed” group was created to include 14 cases presenting the co-occurrence of both rp-ND and non-neurodegenerative etiologies. Overall, 51 subjects received a neuropathological diagnosis. The remaining 162 cases tested negative at the second-generation prion RT-QuIC assay, and the attribution of a high probable clinical diagnosis (i.e., to a particular diagnostic group) was achieved by interpreting clinical, laboratory (e.g., positivity for autoantibodies targeting CNS antigens, positive α-synuclein RT-QuIC assay, CSF biomarker profile suggestive of Alzheimer’s disease), and imaging data in light of the most recent RPD diagnostic algorithms (11,12). Rp-ND patients with a probable clinical diagnosis were classified according to the current diagnostic criteria (13–17). We added the category of AD plus Dementia with Lewy bodies (DLB) to include 5 subjects fulfilling the diagnostic criteria for probable DLB and also exhibiting an AD CSF biomarker profile (14,18). Details regarding the etiologies of all np-RPD cases are presented in Supplementary table 1.

**Supplementary table 1** – np-RPD cohort diagnostic categories

|  | **Whole cohort (n = 213)** | | **Ng cohort (n = 93)** | |
| --- | --- | --- | --- | --- |
| **np-RPD** | Pathological (n = 51) | Clinical  (n = 162) | Pathological  (n = 17) | Clinical  (n = 76) |
| **Non-neurodegenerative** | 28 | 70 | 10 | 33 |
| - Inflammatory | 10 | 39 | 3 | 19 |
| - Epilepsy | - | 3 | - | 1 |
| - Toxic-Metabolic | 2 | 12 | 1 | 5 |
| - Neoplastic | 8 | 2 | 5 | - |
| - Vascular | 8 | 11 | 1 | 8 |
| - Psychiatric | - | 3 | - | - |
| **rp-ND** | 20 | 81 | 5 | 40 |
| - AD | 11 | 62 | 3 | 29 |
| - AD + DLB | 3 | 2 | - | 2 |
| - DLB | 6 | 10 | 2 | 5 |
| - CBS | - | 2 | - | 2 |
| - FTD | - | 4 | - | 2 |
| - PSP | - | 1 | - | - |
| **Mixed** | 3 | 11 | 2 | 3 |

Abbreviations: AD, Alzheimer’s disease; CBS, corticobasal syndrome; DLB, dementia with Lewy bodies; FTD, frontotemporal dementia; Ng, neurogranin; np-RPD, non-prion rapidly progressive dementia; PSP, progressive supranuclear palsy; rp-ND, neurodegenerative np-RPD.

**Supplementary table 2** - Distribution of biomarkers levels in the subgroups of the CJD cohort

| **Diagnostic group** | **N** | **CSF t-tau (pg/mL)** | **CSF 14-3-3 (AU)** | **CSF NfL (pg/mL)** | **CSF p-tau (pg/mL)** |
| --- | --- | --- | --- | --- | --- |
| **sCJD^a^** | 183 | 3259 (1970-8182) | 50500 (27200-105000) | 7239 (3575-11687) | 58 (39-78) |
| sCJD MM(V)1 | 71 | 4956 (3004-10180) | 65500 (39250-137500) | 4871 (2876-7890) | 43 (33-62) |
| sCJD VV2 | 43 | 9195 (5001-13300) | 109000 (74900-171000) | 12000 (8750-15800) | 72 (54-105) |
| sCJD MV2K | 53 | 1688 (1267-2397) | 23100 (16500-33400) | 7357 (3541-10138) | 61 (43-79) |
| sCJD MM(V)2C | 12 | 1382 (1011-4117) | 21250 (11600-44050) | 3889 (1868-10438) | 68 (42-89) |
| sCJD MM2T | 2 | 633, 2787 | 13300, 58600 | 6050, 28550 | 20, 47 |
| sCJD VV1 | 2 | 3325, 3790 | 68200, 37900 | 15900, 11100 | 49, 31 |
| **gCJD^b^** | 37 | 3065 (1816-6735) | 54300 (25800-95800) | 4544 (2747-6200) | 44 (32-63) |
| M1 | 31 | 4236 (2477-7647) | 70300 (37300-98900) | 4664 (3441-6335) | 51 (33-68) |
| M “i” | 2 | 763, 952 | 13200, 15800 | 3290, 14750 | 48, 30 |
| M2T | 3 | 120, 288, 271 | 2680, 6048, 4545 | 1881, 5150, 2618 | 24, 19, 19 |
| V1 | 1 | 1816 | 39200 | 1717 | 44 |

Abbreviations: CSF, cerebrospinal fluid; gCJD, genetic Creutzfeldt-Jakob disease; NfL, neurofilament light chain; p-tau, phospho-tau181; sCJD, sporadic Creutzfeldt-Jakob disease; t-tau, total tau.

^a^Both patients with a definite diagnosis of a specific subtype and patients with a probable diagnosis and a high level of certainty for a given subtype are included.

^b^M1 group includes 13 gCJD E200K-129M, 1 gCJD V203I-129M and 17 gCJD V210I-129M; M “i” group includes 2 gCJD E200K-129M; M2T group includes 3 fatal familial insomnia (FFI) (gCJD D178N-129M); V1 group includes 1 gCJD D178N-129V

Biomarker data are presented as median (IQR)

**Supplementary table 3** - Associations of t-tau, 14-3-3, and NfL CSF levels with survival time in the whole CJD cohort and after stratification according to the disease subtype

| **Diagnostic group and biomarker** | | **Survival time** | **Univariate Cox regression** | | **Multivariate Cox regression^a^** | |
| --- | --- | --- | --- | --- | --- | --- |
|  |  | **Median ± IQR (months)** | **HR (95% CI)** | **P value** | **HR (95% CI)** | **P value** |
| **Whole CJD cohort** | | | | | | |
| **t-tau**  **(N = 215)** | Continuous value | 5.0 (2.8-12.0) | **1.73 (1.49-2.02)** | **<.001** | **1.63 (1.35-1.97)** | **<.001** |
|  | Low tertile | 12.6 (6.0-18.0) | Ref | Ref | Ref | Ref |
|  | Mid tertile | 4.0 (2.5-9.0) | **1.97 (1.41-2.75)** | **<.001** | **1.59 (1.10-2.28)** | **.012** |
|  | High tertile | 4.0 (2.5-5.5) | **3.27 (2.28- 4.68)** | **<.001** | **2.78 (1.82-4.24)** | **<.001** |
| **14-3-3**  **(N = 215)** | Continuous value | 5.0 (2.8-12.0) | **1.72 (1.46-2.02)** | **<.001** | **1.70 (1.38-2.09)** | **<.001** |
|  | Low tertile | 12.0 (6.0-16.0) | Ref | Ref | Ref | Ref |
|  | Mid tertile | 4.0 (2.5-12.0) | **1.62 (1.16-2.26)** | **.004** | **1.51 (1.06-2.16)** | **.021** |
|  | High tertile | 3.7 (2.5-5.5) | **3.09 (2.16-4.40)** | **<.001** | **3.24 (2.07-5.07)** | **<.001** |
| **NfL**  **(N = 215)** | Continuous value | 5.0 (2.8-12.0) | **1.27 (1.09-1.49)** | **.002** | **1.25 (1.05-1.49)** | **.012** |
|  | Low tertile | 5.0 (2.5-15.0) | Ref | Ref | Ref | Ref |
|  | Mid tertile | 4.0 (2.5-8.6) | **1.57 (1.13-2.20)** | **.007** | 1.32 (0.93-1.88) | .112 |
|  | High tertile | 6.0 (3.8-11.2) | **1.48 (1.06-2.07)** | **.020** | **1.45 (1.01-2.08)** | **.040** |
| **Typical CJD^b^** | | | | | | |
| **t-tau**  **(N = 100)** | Continuous value | 2.8 (2.1-4.0) | **1.31 (1.00-1.73)** | **.050** | **1.42 (1.07-1.88)** | **.013** |
|  | Low tertile | 3.1 (2.4-4.0) | Ref | Ref | Ref | Ref |
|  | Mid tertile | 2.8 (2.1-4.0) | 0.89 (0.50-1.57) | .692 | 0.95 (0.53-1.68) | .861 |
|  | High tertile | 2.5 (2.1-3.3) | 1.19 (0.67-2.11) | .544 | 1.49 (0.83-2.68) | .181 |
| **14-3-3**  **(N = 100)** | Continuous value | 2.8 (2.1-4.0) | 1.21 (0.89-1.63) | .206 | **1.43 (1.05-1.95)** | **.022** |
|  | Low tertile | 3.0 (2.4-4.0) | Ref | Ref | Ref | Ref |
|  | Mid tertile | 2.5 (2.0-4.0) | 0.85 (0.48- 1.49) | .580 | 0.83 (0.48-1.47) | .545 |
|  | High tertile | 2.5 (2.1-3.4) | 1.14 (0.65-1.99) | .643 | 1.52 (0.85-2.72) | .150 |
| **NfL**  **(N = 100)** | Continuous value | 2.8 (2.1-4.0) | **1.48 (1.13-1.93)** | **.004** | **1.49 (1.11-2.00)** | **.007** |
|  | Low tertile | 3.0 (2.3-4.0) | Ref | Ref | Ref | Ref |
|  | Mid tertile | 2.5 (2.0-3.5) | 1.54 (0.98-2.43) | .056 | 1.21 (0.76-1.94) | .409 |
|  | High tertile | 2.5 (2.4-4.0) | **1.91 (1.14-3.40)** | **.015** | **2.14 (1.20-3.82)** | **.010** |
| **Slower CJD^c^** | | | | | | |
| **t-tau**  **(N = 115)** | Continuous value | 11.0 (6.0-16.0) | **1.69 (1.38-2.08)** | **<.001** | 1.15 (0.85-1.54) | <.347 |
|  | Low tertile | 15.0 (12.0-24.0) | Ref | Ref | Ref | Ref |
|  | Mid tertile | 14.7 (9.0-19.0) | **1.60 (1.02-2.50)** | **.038** | 1.09 (0.64-1.87) | .738 |
|  | High tertile | 5.5 (4.7-6.6) | **3.93 (2.41-6.43)** | **<.001** | 1.67 (0.81-3.45) | <.161 |
| **14-3-3**  **(N = 115)** | Continuous value | 11.0 (6.0-16.0) | **1.69 (1.35-2.11)** | **<.001** | 0.98 (0.71-1.36) | .943 |
|  | Low tertile | 15.0 (10.0-18-0) | Ref | Ref | Ref | Ref |
|  | Mid tertile | 14.2 (11.2-21.2) | 1.26 (0.81-1.96) | .295 | 0.84 (0.46-1.51) | .563 |
|  | High tertile | 5.5 (4.9-6.6) | **3.97 (2.36-6.68)** | **<.001** | 0.95 (0.40-2.23) | .913 |
| **NfL**  **(N = 115)** | Continuous value | 11.0 (6.0-16.0) | **1.66 (1.33-2.08)** | **<.001** | **1.41 (1.07-1.84)** | **.012** |
|  | Low tertile | 16.0 (11.0-25.0) | Ref | Ref | Ref | Ref |
|  | Mid tertile | 9.0 (5.6-14.9) | **2.15 (1.29-3.57)** | **.003** | 1.30 (0.75-2.28) | .343 |
|  | High tertile | 7.0 (5.1-13.6) | **2.56 (1.61-4.09)** | **<.001** | **1.73 (1.01-2.95)** | **.042** |

Abbreviations: CI, confidence interval; CSF, cerebrospinal fluid; gCJD, genetic Creutzfeldt-Jakob disease; HR, hazard ratio; IQR, interquartile range; NfL, neurofilament light chain; Ref, reference; sCJD, sporadic Creutzfeldt-Jakob disease; t-tau, total tau.

^a^All multivariate Cox regression analyses included codon 129 genotype, age at LP and time from onset to sample collection as covariates.

^b^Includes sCJD MM(V)1 and gCJD M1.

^c^Includes sCJD VV2, sCJD MV2K, sCJD MM(V)2C, sCJD MM2T, sCJD VV1, gCJD M“i“, gCJD M2T, and gCJD V1.

Bold values indicate statistically significant hazard ratios.

**Supplementary figure 1** – t-tau, 14-3-3, and NfL prognostic performance


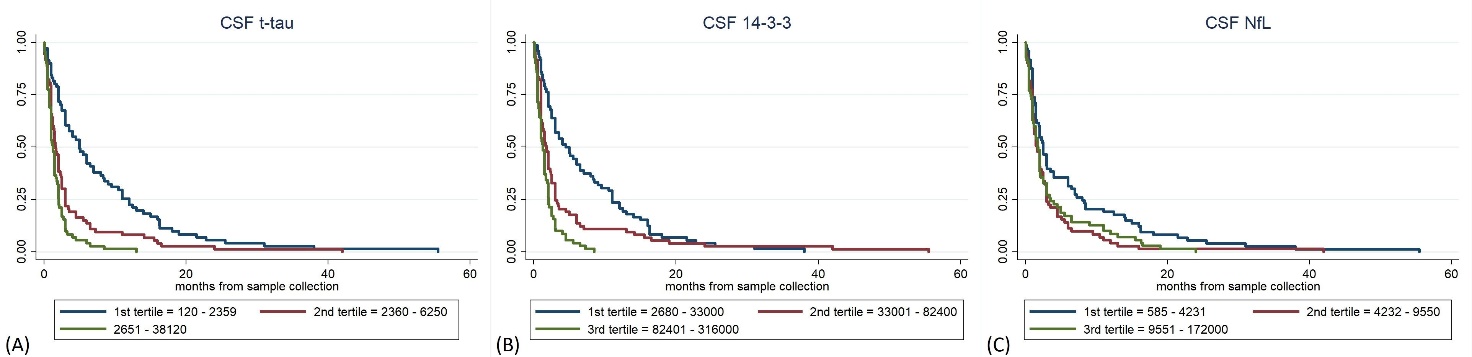


Prognostic value of CSF t-tau (A), 14-3-3 (B), and NfL (C). Survival curves in patients of the whole CJD cohort according to the values of CSF biomarkers.

CSF, cerebrospinal fluid; NfL, neurofilament light chain; t-tau, total tau

**Supplementary figure 2** – Correlation analysis between SNAP-25 or Ng levels and disease stages


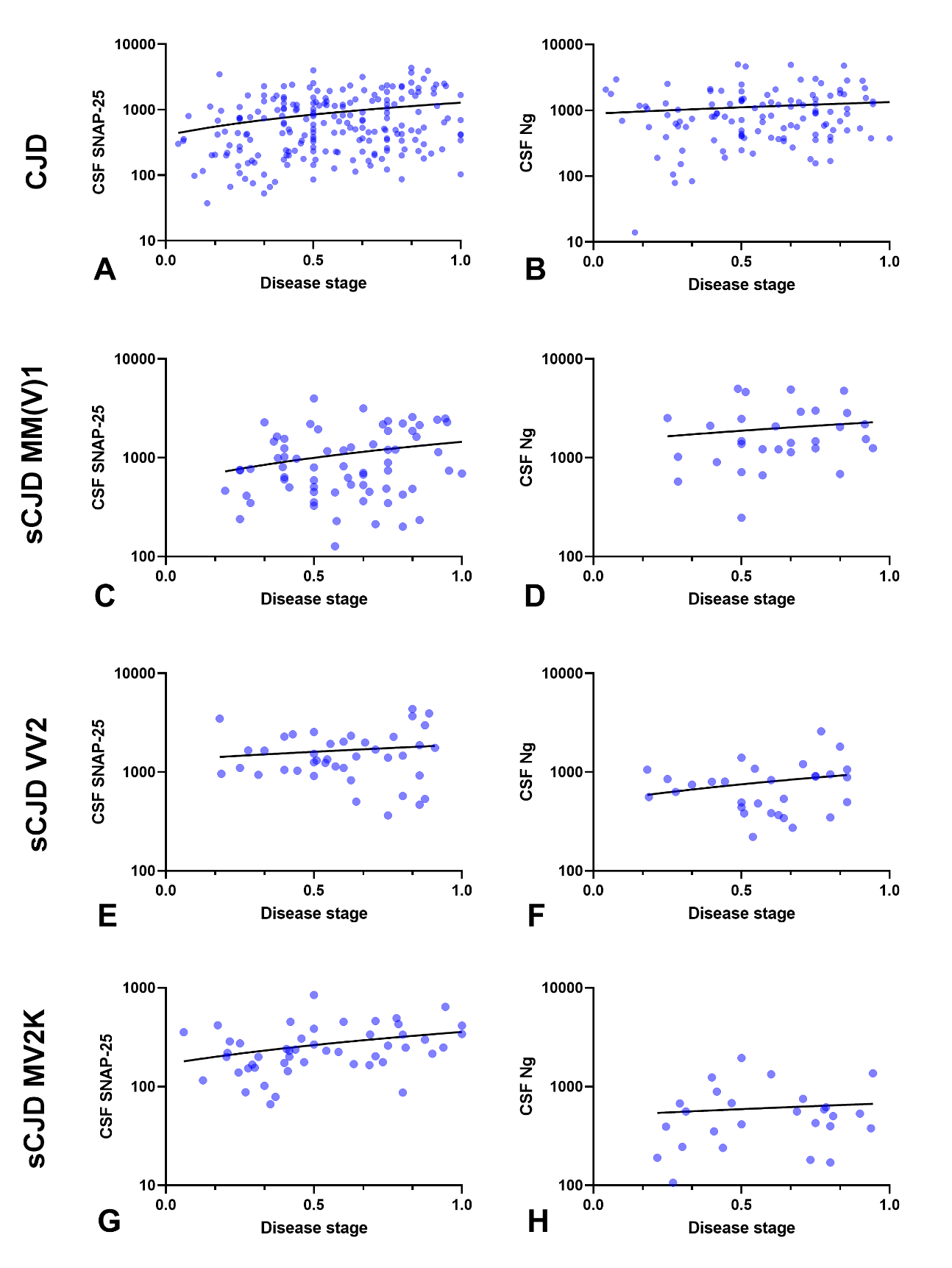


Correlation analysis of SNAP-25 and Ng levels with disease stage in the whole CJD cohort (A-B), sCJD MM(V)1 (C-D), sCJD VV2 (E-F), sCJD MV2K (G-H). Correlation analysis was performed using Spearman’s rank correlation coefficient. See the main text for significant P-values. CSF SNAP-25 and Ng values are expressed in a logarithmic scale.

CJD, Creutzfeldt-Jakob disease; CSF, cerebrospinal fluid; Ng, neurogranin; SNAP-25, synaptosomal-associated protein 25

**Supplementary references**

1. Mastrangelo A, Baiardi S, Zenesini C, Poleggi A, Mammana A, Polischi B, et al. Diagnostic and prognostic performance of CSF α‐synuclein in prion disease in the context of rapidly progressive dementia. Alz & Dem Diag Ass & Dis Mo [Internet]. 2021 Jan [cited 2022 Sep 28];13(1). Available from: https://onlinelibrary.wiley.com/doi/10.1002/dad2.12214

2. Abu-Rumeileh S, Halbgebauer S, Steinacker P, Anderl-Straub S, Polischi B, Ludolph AC, et al. CSF SerpinA1 in Creutzfeldt-Jakob disease and frontotemporal lobar degeneration. Ann Clin Transl Neurol. 2020 Feb;7(2):191–9.

3. Lattanzio F, Abu-Rumeileh S, Franceschini A, Kai H, Amore G, Poggiolini I, et al. Prion-specific and surrogate CSF biomarkers in Creutzfeldt-Jakob disease: diagnostic accuracy in relation to molecular subtypes and analysis of neuropathological correlates of p-tau and Aβ42 levels. Acta Neuropathol. 2017 Apr;133(4):559–78.

4. Parchi P, Giese A, Capellari S, Brown P, Schulz-Schaeffer W, Windl O, et al. Classification of sporadic Creutzfeldt-Jakob disease based on molecular and phenotypic analysis of 300 subjects. Annals of neurology. 46(2):224-233.

5. Zerr I, Kallenberg K, Summers DM, Romero C, Taratuto A, Heinemann U, et al. Updated clinical diagnostic criteria for sporadic Creutzfeldt-Jakob disease. Brain. 2009 Oct 1;132(10):2659–68.

6. Parchi P, de Boni L, Saverioni D, Cohen ML, Ferrer I, Gambetti P, et al. Consensus classification of human prion disease histotypes allows reliable identification of molecular subtypes: an inter-rater study among surveillance centres in Europe and USA. Acta Neuropathol. 2012 Oct;124(4):517–29.

7. Baiardi S, Magherini A, Capellari S, Redaelli V, Ladogana A, Rossi M, et al. Towards an early clinical diagnosis of sporadic CJD VV2 (ataxic type). J Neurol Neurosurg Psychiatry. 2017 Sep;88(9):764–72.

8. Abu-Rumeileh S, Capellari S, Stanzani-Maserati M, Polischi B, Martinelli P, Caroppo P, et al. The CSF neurofilament light signature in rapidly progressive neurodegenerative dementias. Alz Res Therapy. 2018 Dec;10(1):3.

9. Abu-Rumeileh S, Baiardi S, Polischi B, Mammana A, Franceschini A, Green A, et al. Diagnostic value of surrogate CSF biomarkers for Creutzfeldt–Jakob disease in the era of RT-QuIC. J Neurol. 2019 Dec;266(12):3136–43.

10. Rossi M, Kai H, Baiardi S, Bartoletti-Stella A, Carlà B, Zenesini C, et al. The characterization of AD/PART co-pathology in CJD suggests independent pathogenic mechanisms and no cross-seeding between misfolded Aβ and prion proteins. Acta Neuropathol Commun. 2019 Apr 8;7(1):53.

11. Hermann P, Zerr I. Rapidly progressive dementias — aetiologies, diagnosis and management. Nat Rev Neurol. 2022 Jun;18(6):363–76.

12. Geschwind MD. Rapidly Progressive Dementia. Continuum (Minneap Minn). 2016;22(2):510–37.

13. Dubois B, Feldman HH, Jacova C, Hampel H, Molinuevo JL, Blennow K, et al. Advancing research diagnostic criteria for Alzheimer’s disease: the IWG-2 criteria. The Lancet Neurology. 2014 Jun;13(6):614–29.

14. McKeith IG, Boeve BF, Dickson DW, Halliday G, Taylor JP, Weintraub D, et al. Diagnosis and management of dementia with Lewy bodies: Fourth consensus report of the DLB Consortium. Neurology. 2017 Jul 4;89(1):88–100.

15. Armstrong MJ, Litvan I, Lang AE, Bak TH, Bhatia KP, Borroni B, et al. Criteria for the diagnosis of corticobasal degeneration. Neurology. 2013 Jan 29;80(5):496–503.

16. Höglinger GU, Respondek G, Stamelou M, Kurz C, Josephs KA, Lang AE, et al. Clinical diagnosis of progressive supranuclear palsy: The movement disorder society criteria: MDS Clinical Diagnostic Criteria for PSP. Mov Disord. 2017 Jun;32(6):853–64.

17. Rascovsky K, Hodges JR, Knopman D, Mendez MF, Kramer JH, Neuhaus J, et al. Sensitivity of revised diagnostic criteria for the behavioural variant of frontotemporal dementia. Brain. 2011 Sep;134(9):2456–77.

18. Jack CR, Bennett DA, Blennow K, Carrillo MC, Dunn B, Haeberlein SB, et al. NIA‐AA Research Framework: Toward a biological definition of Alzheimer’s disease. Alzheimer’s &amp; Dementia. 2018 Apr;14(4):535–62.
